# Brief cognitive battery in the diagnosis of mild Alzheimer’s disease in subjects with medium and high levels of education

**DOI:** 10.1590/S1980-57642008DN10100006

**Published:** 2007

**Authors:** Ricardo Nitrini, Paulo Caramelli, Claudia Sellitto Porto, Helenice Charchat-Fichman, Ana Paula Formigoni, Maria Teresa Carthery-Goulart, Carla Otero, João Carlos Prandini

**Affiliations:** 1 MD, PhD, Behavioral and Cognitive Neurology Unit, Department of Neurology, and Cognitive Disorders Reference Center (CEREDIC). Hospital das Clínicas of the University of São Paulo School of Medicine.; 2 MD, PhD, Behavioral and Cognitive Neurology Unit Faculty of Medicine, Federal University of Minas Gerais.; 3 PhD, Behavioral and Cognitive Neurology Unit, Department of Neurology, and Cognitive Disorders Reference Center (CEREDIC). Hospital das Clínicas of the University of São Paulo School of Medicine.

**Keywords:** Alzheimers disease, dementia, diagnosis, education, brief cognitive battery, neuropsychological tests, doença de Alzheimer, demência, diagnóstico, educação, bateria cognitiva breve, testes neuropsicológicos

## Abstract

There has been an increasing trend to utilize short cognitive batteries for the diagnosis of dementia. Most of these batteries have been designed in countries with high standards of education and are less suitable for populations with low levels of education.We developed a battery that has been previously shown to be highly accurate in the diagnosis of dementia in individuals with low levels of education. The accuracy of this battery for patients with higher levels of education is unknown.

**Objectives:**

To evaluate the accuracy of a brief cognitive battery in the diagnosis of Alzheimer’s disease (AD) in subjects with medium and high levels of schooling, and to develop a mathematical model that includes the most discriminative tests.

**Methods:**

Seventy-three mildly demented patients with probable AD and 94 control subjects were evaluated. Sixty patients and 60 controls were randomly selected to generate a mathematical model including the most discriminative tests of the battery using logistic regression. The model was back-tested for the remaining sample of patients and controls.

**Results:**

Delayed recall, learning and category fluency tests were included in a mathematical model that obtained an area of 0.917 in the ROC curve in the back-testing. Inter-rater reliabilities of these tests were high (kappa>0.8).

**Conclusions:**

This model showed a high accuracy for the diagnosis of mild AD in patients with medium and high educational levels. Future studies with more heterogeneously educated individuals are necessary to investigate whether the educational level (number of years at school) should also be included in the model.

## Abstract (pt)

Existe crescente tendência de se utilizarem baterias cognitivas breves no diagnóstico de demência. A maioria destas tem sido idealizada em países com altos níveis de escolaridade e é menos adequada para populações com baixa escolaridade. Desenvolvemos uma bateria que tem demonstrado alta acurácia no diagnóstico de demência em indivíduos com baixa escolaridade, mas a acurácia em indivíduos com escolaridade alta ainda não é conhecida.

**Objetivos:**

Avaliar a acurácia de uma bateria cognitiva breve no diagnóstico de doença de Alzheimer (DA) em indivíduos com escolaridade média ou alta, e desenvolver um modelo matemático que inclua os testes mais discriminativos.

**Métodos:**

Setenta e três pacientes com demência leve causada por DA provável e 94 controles foram avaliados com a bateria cognitiva breve. Sessenta pacientes e 60 controles foram selecionados aleatoriamente para gerar um modelo matemático com os testes mais discriminativos, empregando regressão logística. Este modelo foi retro-testado na amostra remanescente de pacientes e controles.

**Resultados:**

Os testes de memória tardia, aprendizado e fluência verbal foram incluídos no modelo matemático que obteve área sob a curva ROC de 0,917 no retro-teste. A confiabilidade inter-examinadores destes testes foi alta (kappa>0.8).

**Conclusões:**

Este modelo apresentou alta acurácia no diagnóstico de demência leve na DA em sujeitos com escolaridade média ou alta. Novos estudos com população com maior heterogeneidade educacional são necessários para investigar se os anos de escolaridade também devem ser incluídos no modelo.

There has been an increasing trend to utilize short cognitive batteries for the diagnosis of dementia. These batteries of neuropsychological tests may be used in epidemiological studies or in the busy clinical settings by specialists as well as by primary care physicians. Most, however, have been developed and validated in developed countries^1-4^, thus not reflecting the reality in developing and underdeveloped countries, where low education and even illiteracy are common, especially among the elderly. Particularly, batteries designed in developed countries usually include items that rely on reading or writing abilities, which are less suitable for populations with low levels of education.

For several years we have been working on a cognitive battery developed to evaluate subjects independent of their educational level, and thus applicable to populations with heterogeneous educational background^5-7^. In a previous study, we showed that the performance of illiterate non-demented elderly subjects was higher in the delayed recall test of our brief battery than in the Consortium to establish a registry for Alzheimers disease (CERAD) battery^6^. Subsequently, we showed that the delayed recall test of our battery was also more accurate than the equivalent test of the CERAD battery for diagnosing dementia in illiterates from an elderly cohort^7^.

On the other hand, a battery designed for diagnosing cognitive impairment in subjects which are illiterate or have low levels of education may not be applicable for those with high educational levels.

Thus, this study aims at evaluating the accuracy of this brief cognitive battery in the diagnosis of mild dementia caused by Alzheimer’s disease (AD) in patients with medium and high levels of schooling, as well as to ascertain the most discriminative tests, and develop a mathematical model that could be applicable in clinical practice.

## Methods

### Subjects

All patients with mild dementia due to probable AD according to NINCDS-ADRDA criteria^8^, who were evaluated by two senior neurologists (RN and PC) and submitted to a comprehensive neuropsychological battery by two psychologists (CSP and HCF) were included. All had laboratory examinations and CT or MRI of the head to exclude other causes of dementia. For the diagnosis of dementia, the neuropsychological battery consisted of the Mini-mental state examination – MMSE^9,10^, the Dementia Rating Scale – DRS^11,12^, visual reproduction^13^, Rey complex figure^14^, logical memory^13^, Rey auditory verbal learning test^14,15^, block design^16^, Hooper visual organization test^17^, Raven’s progressive matrices^14^, Boston naming test^18,19^, trail making test (A and B versions), Stroop color test and phonemic verbal fluency (F.A.S. test)^14^. Patients were diagnosed as mildly demented when the DRS global scores fell within the 110 to 122 range^12^. Seventy-three patients were included.

Ninety-four subjects were included in the healthy control group. They were volunteers recruited among spouses or friends of the patients or drawn from senior citizen groups. A semi-structured questionnaire was used to exclude those with memory or cognitive problems. Subjects who were using medications that could have interfered in the cognitive performance were excluded, as well as those with a history of alcoholism or uncontrolled chronic diseases. Normal control subjects were evaluated with the MMSE and the DRS.

Demographic data of patients and control subjects are shown in Table 1.

**Table 1:** Demographic data and mean Mini-Mental State Examination (MMSE) and Dementia Rating Scale (DRS) scores of patients with Alzheimer's disease (AD) and control subjects.

	Controls (N=94)	AD patients (N=73)	p
Age	71.1(6.4)	73.3 (7.4 )	0.050
Gender	37 M/57 W	29 M/44W	0.911
Years of schooling (mean)	10.8 (4.5 )	11.1 (4.7 )	0.629
MMSE mean (SD)	27.4 (1.6)	23.2(3.0)	<0.001
DRS mean (SD)	135.6 (6.1)	116.2 (3.9)	<0.001

M, men;W, women.

### Procedures

The brief cognitive battery was administered to all patients and controls, through the display of a sheet of paper in which 10 simple objects were presented as line drawings (shoe, house, comb, key, airplane, bucket, turtle, tree, spoon and book)^5,6^. First, the subject was asked to name each drawing. The examiner scored the number of correct identifications and naming of the drawn objects. If the subject was able to properly *name* the drawn objects, the identification was also deemed correct. However, if the naming was incorrect, this could indicate either failure in identification or in naming itself, although this differentiation was usually straightforward. For instance, if the patient named the airplane a fish, this was clearly an identification problem.When *naming* was incorrect or not remembered, the examiner provided the correct names. Immediately after naming all objects, the sheet of paper was removed from view, and the subject was asked to recall the drawings (incidental memory). The sheet of paper was then shown again for about 30 seconds, the patient was explicitly asked to memorize the objects, and recall was requested immediately after presentation. This latter procedure was performed twice, leading firstly to a score of immediate memory and then to a score of what was called learning test. After an interference period in which the category fluency test (number of animals in one minute) and the clock drawing test – CDT^20^ were performed, subjects were then asked to recall as many items as they could, for no more than one minute, where this constituted the delayed recall test. As a final procedure, a sheet of paper with 20 drawings, including the 10 previously presented along with 10 added distractors was displayed. The score of this recognition test was calculated by subtracting the number of wrong from the correct responses. To summarize, the brief cognitive battery consists of the identification, naming, incidental memory, immediate memory, learning, delayed recall and recognition of 10 simple drawings associated with the category fluency test and CDT.

In order to verify the reliability of the battery, 22 elderly controls were tested in the presence of two examiners (RN and PC) who scored the subject independently. The time taken to accomplish the battery was recorded for each of these 22 subjects. Sixty patients and 60 controls were randomly selected to generate a mathematical model using logistic regression with the most discriminative tests of the battery. The generated model was backtested for the remaining sample of patients (n=13) and controls (n=34).

ROC (receiver operator characteristic) curves were used to evaluate the accuracy of each test. Inter-rater reliability was evaluated using kappa values.

Comparisons between means of demographic data were performed through the t test or chi-square test when appropriate. For comparisons of test scores of patients and controls, the Mann-Whitney test was used. The value of significance accepted was 0.05. The software package SPSS for Windows 10.0 was used for the statistical analysis.

This study was approved by the Ethics Committee of the Hospital das Clínicas of the University of São Paulo School of Medicine. All subjects (or a relative, if necessary) were informed about the study prior to the evaluation, and informed consent was given.

## Results

Patients and controls did not differ regarding the identification of the simple drawings (p=0.20), while all other tests of the battery were different for the two groups. Although naming was also different, 83.6% of the patients were able to correctly name the 10 drawings.When the whole sample was included, the delayed recall was the most discriminative test to correctly identify patients and controls. The areas under the curves (AUC), cut-off scores, sensitivities and specificities are shown in Table 2.

**Table 2:** Cut-off scores of the most discriminative tests for the whole sample.

	Area under the curve (95% CI)	Cut-off scores	Sensitivity (%)	Specificity (%)
Delayed recall test	0.931 (0.894;0.968)	<6	82.2	90.4
Learning test	0.903 (0.857;0.948)	<7	90.4	74.5
Verbal fluency	0.839 (0.778;0.900)	<15	83.6	67.0
Immediate memory	0.890.(0.839;0.941)	<7	90.4	77.7
Incidental memory	0.866 (0.812;0.920)	<5	81.7	75.5
Recognition	0.903 (0.857;0.948)	<8	90.4	74.5
Clock-drawing	0.769 (0.692;0.845)	<9	77.8	72.3

In the randomly selected sample of 60 patients and 60 controls, multivariate analysis selected the delayed recall, learning and category fluency tests to generate the model below:

$$\mathrm{Score}=100x\frac{\exp\left(12.642-\left(0.640\;\mathrm{dr}\right.\right)-\left(0.794\;\mathrm{lt}\right)-\left(0.255\;\mathrm{cf}\right)}{1+\exp\left(12.642-\left(0.640\;\mathrm{dr}\right.\right)-\left(0.794\;\mathrm{lt}\right)-\left(0.255\;\mathrm{cf}\right)}$$

where dr=delayed recall test, lt=learning test and cf= category fluency test, in which the higher the score, the higher the probability of dementia. In the back-testing, the model obtained an AUC-ROC of 0.917. The kappa values of the inter-rater reliability were 1.0 for the learning test, 0.913 for the delayed recall test and 0.846 for the category fluency test. The mean time to accomplish the battery was 487.7 (±87.6) seconds, with a median of 470 seconds.

## Discussion

This study showed that this brief cognitive battery is highly accurate for the diagnosis of mild dementia in AD patients with medium-high educational levels, confirming the findings obtained in illiterate and low educated individuals. The inter-rater reliability was high, and the time to accomplish, about 8 minutes in elderly individuals, is not long considering the many cognitive functions that are evaluated.

A comparative advantage of this battery in relation, for instance, to the CERAD, is its similar procedural applicability and validity, regardless of the educational level of the subject or his or her level of mastering of the official language of their country of residence. Because of the possibility of using this battery for the evaluation of subjects with wide educational background, we decide to name it Brief Cognitive Battery – Unbiased by Education (BCB-Edu) to emphasize this information.

Other brief batteries consisting of simple and rapid tests have been proposed for dementia diagnosis. The Mini-Cog is based on the delayed recall of three words and on the CDT, having been used as a screening test^3^. In a previous study, we found that non-demented illiterate subjects have great difficulty in performing the CDT^6,20^. While non-demented literate subjects obtained a median of 9 on the CDT, the median for illiterates was 2.56. At least for our population, it is probable that the use of this combination is not appropriate.

Another interesting test is the Memory Impairment Screen, which is based on the delayed recall of four written words that are encoded with semantic cues^21^. However, its dependency on reading ability hampers its use for low educated subjects or for immigrants unable to read in the official language of their country of residence.More recently, it has been reported that the combination of the recall of a five-item name and address and category verbal fluency (animals in one minute) had high sensitivity and specificity for the diagnosis of dementia in a highly educated study sample^22^.

Delayed recall tests are considered the best single tests to diagnose AD^23,24^, while word generation tasks also have shown high specificity and sensitivity for the diagnosis of dementia^25,26^. The combination of the two types of tests, as proposed by Kilada et al.^22^ and by the BCB-Edu, is likely to prove very useful for the diagnosis of AD and other types of dementia. However, the animal fluency test is influenced by education, particularly by illiteracy, making it necessary to use different cut-off scores for different educational levels. For instance, while one study reported that the best cut-off score to differentiate AD from normal controls was 15 in a study sample with high education levels^25^, a recent Brazilian study reported that the best cut-off for diagnosing dementia in illiterate subjects is 9^27^.

A few points deserve consideration. Firstly, as the time to accomplish the BCB-Edu is higher than the most recently proposed tests^3,21,22^, could only the three most discriminative tests be used rather than all nine items scored in the battery (identification, naming, incidental memory, immediate memory, learning task, delayed recall, recognition, category fluency and CDT)? As it is clear from the application procedures, that the identification, naming, incidental memory, and immediate memory steps are pre-requirements to the learning and delayed recall steps, they cannot be eliminated.

It is possible that the recognition test and CDT could be eliminated, reducing the procedure to about five minutes.

However, as the clock-drawing, together with the category fluency test, is included between the phase of memorizing the 10 figures and their delayed recall, it remains to be decided whether the exclusion of the CDT would impact the delayed recall score. In a recent study, the scores in a delayed recall test were not different when the time delays between encoding and recall were either two or four minutes^22^, which makes plausible the suggestion that the CDT be removed from the BCB-edu.

We were able to develop a mathematical model for the diagnosis of dementia. However, it is necessary to study whether this model is consistent when individuals with low levels of education or illiterates are included. Future studies should investigate whether the educational level (number of years at school) should be included in the model. Also, it will be necessary to develop a practical instrument for calculating the final score at the clinic, possibly using a palm-top or laptop.

Finally, as suggested above, by not relying on auditory reading or written material, BCB-Edu may allow reliable cognitive evaluation of those elderly individuals who are functional illiterates, or immigrants unable to read or to write in the official language of their country of residence, an ever increasing trend in many developed countries^28,29^.

## References

[1] Morris JC, Heyman A, Mohs RC (1989). The Consortium to establish a registry for Alzheimer's disease
(CERAD) Part I. clinical and neuropsychological assessment of Alzheimer's
disease. Neurology.

[2] Meulen EF, Schmand B, van Campen JP (2004). The seven minute screen a neurocognitive screening test highly
sensitive to various types of dementia. J Neurol Neurosurg Psychiatr.

[3] Borson S, Scanlan J, Brush M, Vitaliano P, Dokmak A (2000). The mini-cog a cognitive 'vital signs' measure for dementia
screening in multi-lingual elderly. Int J Geriatr Psychiatr.

[4] Lorentz WJ, Scanlan JM, Borson S (2002). Brief screening tests for dementia. Can J Psychiatr.

[5] Nitrini R, Lefèvre BH, Mathias SC (1994). Neuropsychological tests of simple application for diagnosing
dementia. Arq Neuropsiquiatr.

[6] Nitrini R, Caramelli P, Herrera E Jr (2004). Performance of illiterate and literate nondemented elderly
subjects in two tests of long-term memory. J Int Neuropsychol Soc.

[7] Takada LT, Caramelli P, Fichman HC (2006). Comparison between two tests of delayed recall for the diagnosis
of dementia. Arq Neuropsiquiatr.

[8] McKhann G, Drachman D, Folstein M (1984). Clinical diagnosis of Alzheimer's disease report of the
NINCDSADRDA work group under the auspices of department of health and human
services task force on Alzheimer's disease. Neurology.

[9] Folstein MF, Folstein SE, McHugh PR (1975). "Mini-mental State": a practical method for grading the cognitive
state of patients for the clinician. J Psychiatr Res.

[10] Brucki SMD, Nitrini R, Caramelli P, Bertolucci PHF, Okamoto IH (2003). Sugestões para o uso do Mini-Exame do Estado Mental no
Brasil. Arq Neuropsiquiatr.

[11] Mattis S (1988). Dementia Rating Scale: professional manual.

[12] Porto SC, Charchat-Fichman H, Caramelli P, Bahia VA, Nitrini R (2003). Dementia Rating Scale - DRS - in the diagnosis of patients with
Alzheimer's dementia. Arq Neuropsiquiatr.

[13] Weschler D (1987). Weschler Memory Scale - revised manual.

[14] Spreen O, Strauss E (1998). A compendium of neuropsychological tests: administration, norms, and
commentary.

[15] Diniz LFM, Cruz MF, Torres VM, Consenza RM (2003). O teste de aprendizagem auditivo-verbal de Rey normas para uma
população brasileira. Rev Bras Neurol.

[16] Wechsler D (1993). Test de inteligência para adultos (WAIS). Manual.

[17] (1983). Hooper Visual Organization Test (VOT) Manual.

[18] Kaplan E, Goodglass H, Weintraub S, Segal H (1983). Boston Naming Test.

[19] Mansur LL, Radanovic M, Araujo G de C, Taquemori LY, Greco LL (2006). Boston Naming Test performance of Brazilian population from Sao
Paulo. Pro Fono.

[20] Sunderland T, Hill JL, Mellow AM (1989). Clock drawing in Alzheimer's disease a novel measure of dementia
severity. J Am Geriatr Soc.

[21] Buschke H, Kuslansky G, Katz M, Stewart WF (1999). Screening for dementia with the Memory Impairment
Screen. Neurology.

[22] Kilada S, Gamaldo A, Grant EA, Moghekar A, Morris JC, O'Brien RJ (2005). Brief screening tests for the diagnosis of dementia comparison
with the mini-mental state exam. Alzheimer Dis Assoc Disord.

[23] Welsh K, Butters N, Hughes J, Mohs R, Heyman A (1991). Detection of abnormal memory decline in mild cases of Alzheimer's
disease using CERAD neuropsychological measures. Arch Neurol.

[24] Bertolucci PH, Okamoto IH, Brucki SM, Siviero MO, Toniolo Neto J, Ramos LR (2001). Applicability of the CERAD neuropsychological battery to
Brazilian elderly. Arq Neuropsiquiatr.

[25] Canning SJD, Leach L, Stuss D, Ngo L, Black SE (2004). Diagnostic utility of abbreviated fluency measures in Alzheimer
disease and vascular dementia. Neurology.

[26] Cummings JL (2004). The one-minute mental status examination. Neurology.

[27] Caramelli P, Carthery-Goulart MT, Porto CS, Charchat-Fichman H, Nitrini R Category fluency as a screening test for Alzheimer's disease in
illiterate and literate patients. Alzheimer Dis Assoc Disord.

[28] Gazmararian JA, Baker DW, Williams MV (1999). Health literacy among Medicare enrollees in a managed care
organization. JAMA.

[29] Parker C, Philp I (2004). Screening for cognitive impairment among older people in black
and minority ethnic groups. Age Ageing.

